# Workplace Interventions to Improve Physical Activity, Reduce Sedentary Behavior, and Promote Healthy Diet Among Employees in Low- and Middle- Income Countries: A Systematic Review

**DOI:** 10.21203/rs.3.rs-9730653/v1

**Published:** 2026-07-14

**Authors:** Samina Akhtar, Khadija A. Sumra, Zainab Samad, Gerald S. Bloomfield, Salim S. Virani, Simon Walker, Gerardo Z. Gomez, Aysha Almas

**Affiliations:** Aga Khan University; Medical College, Aga Khan University; Aga Khan University; Duke University; Aga Khan University; University of York; University of York; Aga Khan University

**Keywords:** Workplace interventions, Cardiovascular disease risk factors, Physical activity, Sedentary behavior, Diet, Employees, Developing countries, Low- and middle-income countries

## Abstract

**Background:**

Cardiovascular diseases (CVD) are the leading cause of mortality globally, with a disproportionate burden in low- and middle-income countries (LMIC). The workplace presents a strategic setting for preventive interventions targeting modifiable risk factors like physical inactivity, sedentary behavior, and poor diet among adults. This systematic review aimed to synthesize evidence on the effectiveness and implementation of workplace interventions targeting physical activity, sedentary behavior, or diet among employees in LMICs.

**Methods:**

We conducted a systematic review following PRISMA guidelines. Five databases (PubMed, Scopus, Cochrane CENTRAL, OATD, Google Scholar) were searched for studies published between January 2000 and May 2025. Eligible studies included working adults in LMIC workplaces, assessed interventions targeting at least one CVD-related behavior (physical activity, sedentary behavior, diet), and reported behavioral or cardiometabolic outcomes. Two reviewers independently screened studies, extracted data, and assessed risk of bias. Given the heterogeneity in study designs, interventions, and outcomes, findings were synthesized narratively.

**Results:**

Sixteen studies (11 randomized controlled trials (RCTs)) from six countries (South Africa, Malaysia, Iran, India, Thailand, Mexico) were included. Interventions were heterogeneous: seven (43%) focused on physical activity (PA), five (31%) on reducing sitting time, four (25%) included dietary interventions, embedded within larger multicomponent programs.

Technology-supported interventions (e.g., Stepathlon pedometer programme) increased step counts by +3,519 steps/day. Environmental interventions (sit-stand desks, text prompts) reduced sitting time by 6–66 minutes/day (largest effect: –66 min/day with frequent prompts). Multicomponent programmes that included dietary counselling or canteen modifications improved fruit and vegetable intake (27% to 64%) and reduced fat/calorie consumption (–553 ± 339 kcal). A standalone nutrition education programme also improved dietary knowledge and reduced sweet/snack intake.

Multi-component, theory-informed programmes showed the most consistent improvements: systolic blood pressure reductions up to –10.2 mmHg, weight loss up to –1.8 kg, and total cholesterol reductions up to –0.45 mmol/L (≈17.4 mg/dL). Single-component environmental or technology-only interventions rarely improved biomarkers.

Common implementation challenges included high attrition (15–30%) and declining digital engagement (app use fell from 77% to 31% by month 6). Overall risk of bias was moderate to high, driven by lack of blinding, reliance on self-reported outcomes (60% of studies), and attrition bias.

**Conclusion:**

Workplace interventions in LMICs show promise for improving CVD risk profiles, particularly multicomponent programs, although the number of studies remains limited. Simple modifications in physical activity, sedentary behavior and diet provide a feasible entry point but should be augmented with components designed to increase MVPA.

## Background

Low- and middle-income countries (LMICs) bear a disproportionate burden of cardiovascular disease (CVD) morbidity and mortality. Premature mortality is also rising (18 million deaths before age 70 annually), largely driven by demographic transitions, rapid urbanization, and changes in lifestyle behaviors(1,2). Approximately 79.6% of the global CVD burden is attributable to modifiable risk factors, notably physical inactivity (30%) (3), sedentary behaviour (7–8%) (4), unhealthy diets (30%) (5), tobacco use, air pollution, obesity, and harmful alcohol consumption(6,7).

Physical inactivity and prolonged sedentary time are particularly relevant in the modern workplace, where a growing proportion of the global workforce is engaged in office-based or service occupations that demand extended periods of sitting. Since adults spend nearly one-third of their waking hours at work, the workplace represents a strategic yet underutilized setting for interventions aimed at reducing these risk factors (8).

Penalvo et al. (2021) reported that multicomponent workplace programs included environmental modification, education, self-monitoring, and incentive structures in high-income countries can produce modest but meaningful improvements in physical activity, diet, and selected cardiometabolic outcomes (9). Importantly, digital health approaches (wearables, smartphone apps and text message prompts) have emerged as scalable methods for behavior change and self-monitoring, with promising effects on step counts and sedentary time in diverse populations (10).

Several recent reviews have summarized workplace health promotion, but evidence syntheses often either focus on high-income countries or pool heterogeneous settings, obscuring LMIC-specific insights (11–13). A targeted review of workplace interventions in LMIC is needed to assess intervention characteristics (e.g., face-to-face vs digital; environmental vs educational), theoretical underpinnings, outcome measures (objectively measured steps vs self-report), and reported effects on behavioral (e.g. physical activity, diet) and outcomes. Additionally, understanding issues in implementation, feasibility, adherence, cost, and sustainability is essential to inform scale-up strategies in severely resource-constrained workplaces in LMIC.

This systematic review aims to synthesize the literature on workplace-based interventions delivered in LMICs that target physical activity, sedentary behavior, and/or diet among adults working in offices. Our objectives are to: (1) Describe the types and components of interventions assessed in LMIC workplaces; (2) Report effects of these interventions on behavioral or cardiometabolic outcomes; (3) Summarize intervention characteristics, fidelity, and acceptability where reported; and (4) Identify research gaps and priorities to guide future trials and translation into policy.

## Methods

The review was conducted and reported in accordance with the Preferred Reporting Items for Systemic reviews and Meta-analyses (PRISMA) guidelines (Supplementary file 1) (14). The protocol was registered with the University of York Centre for Reviews and Dissemination PROSPERO database (CRD420251032463).

### Search strategy and inclusion criteria

We included studies based on predetermined criteria according to the Population, Intervention, Comparison, Outcomes, and Study (PICOS) framework, as given in Table 1. For this review, ĽMIC' were defined as all low-income, lower-middle-income, and upper-middle-income economies as per the World Bank classification for the 2024 fiscal year (15). We searched PubMed/MEDLINE, Scopus, Cochrane CENTRAL, Open Access Theses and Dissertations (OATD) and Google Scholar, for studies published in English from January 2000 to May 2025. Search terms combined keywords and MeSH for workplace settings (e.g., “employee”, “workplace”, “office”), behaviours (e.g., “physical activity”, “sedentary behaviour”, “diet”, “nutrition”) and contexts (e.g., “developing countries”, “LMIC”, “Asia”, “Africa”, “Latin America”).

A librarian reviewed the final search strings (see Supplementary File 2 for full search strategy). Reference lists of included studies were hand-searched to identify additional eligible studies. The search strategy was supplemented by inclusion of individual LMIC country names based on the World Bank classification (15) to ensure comprehensive retrieval.

## Data extraction and synthesis

Data extraction and synthesis

Rayyan software was used for data management. Titles/abstracts were screened independently by two reviewers (SA, KS); full texts of potentially eligible papers were retrieved and assessed against inclusion criteria. Disagreements were resolved by discussion. Data extracted included country, setting, study design, sample size, intervention components and duration, delivery mode (face-to-face, digital, environmental), theoretical underpinning, and outcomes (Table 2). As included studies used heterogeneous interventions and outcome measures, we performed a narrative synthesis structured by intervention type, delivery mode, and reported effects.

## Quality assessment

Randomized controlled trials were appraised using the revised Cochrane Risk of Bias tool (RoB 2), which evaluates bias across five domains: bias arising from the randomization process, deviations from intended interventions, missing outcome data, measurement of the outcome, and selection of the reported result. Non-randomized studies were assessed using ROBINS-I, which evaluates bias due to confounding, selection of participants, classification of interventions, deviations from intended interventions, missing data, measurement of outcomes, and selection of reported results, in line with Cochrane guidance (16). Two reviewers (SA, KS) independently rated the risk of bias, with disagreements resolved through consensus. An overall risk of bias judgement was assigned for each study based on the highest level of risk identified across domains, following tool-specific algorithms.

## Results

### Search results and study characteristics

**Figure 1:**
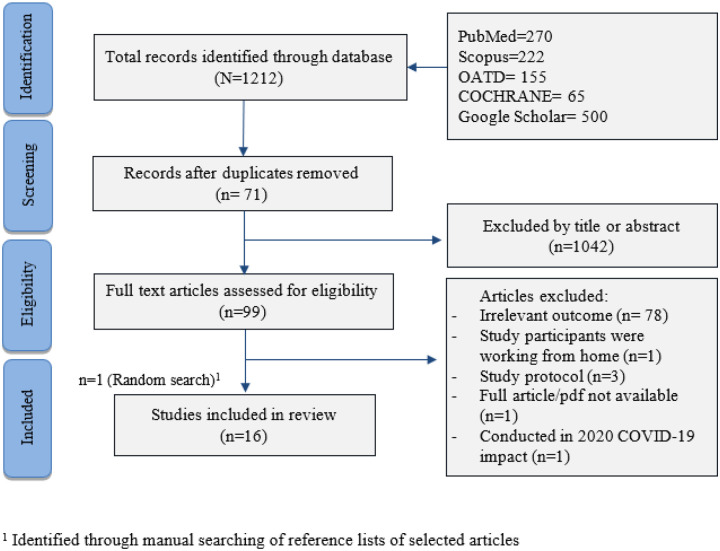
Prisma flow chart

Our search across five databases yielded 1,212 records (Fig. 1). After duplicate removal (n = 71) and title/abstract screening (n = 1,042), 99 full-text articles were assessed for eligibility. The included studies ((n = 16) were published between 2014 and 2025 across six LMIC settings: South Africa (n = 5) (17–21) Malaysia (n = 3) (22–24), Iran (n = 3) (25–27), India (n = 3) (28–30), Thailand (n = 1) (31) and Mexico (n = 1) (32).

Across all included studies, sample sizes ranged from 19 to over 69,000 participants; however, most individual studies enrolled between 60 and 300 employees. Mean age of participants ranged from 27.5 to 42.7 years, and female participation varied from 0% (25) to 100% (27). Office-based and sedentary desk workers were the most frequently studied occupational group, followed by industrial workers and teachers. Study designs included randomized controlled trials (RCTs; n = 11) (17,21,22,25–29), including pilot trials (18,19), and cluster RCTs (31). One RCT only reported on usability of a mobile application (25). Quasi-experimental studies (n = 2) (24,32), single-arm pre-post designs (n = 2) (20,23), and cohort approaches (n = 1) (30) were also included. Intervention duration ranged from six weeks to two years, with follow-up conducted immediately post-intervention in 14 of the 16 studies.

^1^Identified through manual searching of reference lists of selected articles

### Intervention types and components

The interventions exhibited considerable heterogeneity in their approach, ranging from single-component strategies to multi-level programs.

Seven studies (43%) focused on PA interventions, employed diverse strategies, including structured, supervised programs such as High-Intensity Interval Training (HIIT) (23), and Zumba (22). Other approaches utilized pedometers (18), Stepathlon events (30), Fitbits with financial incentives (31), or forming social support groups (22,25).

Five studies (31%) targeted reducing sitting time through sit-to-stand workstations (21), in-person awareness sessions (25), technology-assisted prompts sent via smartphone applications (29), emails (30), and text messages (19).

Four studies (25%) included dietary interventions, embedded within larger multi-component programs featuring individualized dietary counseling (22,24), calorie restriction or environmental nudges in workplace canteens (17). One study implemented a workplace-based nutrition education program to improve dietary practices and cardiometabolic markers (27).

The most complex interventions (n = 6) integrated multi-component programs: PA, diet, and psychological or environmental elements. These programs combined education, supervised exercise, policy changes, and environmental restructuring. Delivery methods were adapted to the context and resources. Face-to-face delivery was used for intensive, supervised exercise or counseling sessions (22,26). Digital/remote methods included smartphone applications (SMART-STEP) (29), pedometers (30), and automated email feedback (18). Hybrid models were also used, blending onsite social support (e.g., group sessions) with digital tools (e.g., Fitbit, text prompts) (25,31).

## Use of theoretical frameworks

The frameworks were included in 43% (n = 7) of studies (TIPPME framework (17), Social-Ecological Model (31); Health Belief Model (24); Self-Determination Theory(29); Feedback Intervention Theory (29); Social Cognitive Theory (19); Theory of Planned Behavior (27); Spence and Lee model (26)). The remaining studies did not explicitly report the use of any theoretical framework.

## Effects on Behavioural and Cardiometabolic Outcomes

Interventions were generally effective at improving short-term behaviour outcomes, though the magnitude and clinical significance varied considerably by intervention type and measurement method.

### Physical activity outcomes:

Significant increase in step counts and light physical activity were observed in pedometer-based programs (e.g., Stepathlon: +3,519 steps/day) (30), social support interventions (25), multicomponent programs (20,26), and technology-assisted prompts (19,28). The lack of improvement in moderate-tovigorous physical activity (MVPA) is notable; however, most interventions were primarily designed to reduce sedentary behaviour or promote light physical activity rather than increase MVPA.

#### Sedentary behavior outcomes

Sedentary behavior (SB) reduction interventions achieved significant decreases in sitting time ranging from 6 to 66 minutes per day (21,25). The largest effect (−66 min/day) was achieved with frequent (every 30 minutes) text message prompts ((21).

#### Dietary outcomes

Positive changes were reported in multi-component programs that included dietary counseling or environmental modifications, such as increased fruit/vegetable consumption (27% to 64%) (20), and reduced caloric/fat intake(22). A nutrition education program based on the Theory of Planned Behavior demonstrated significant improvements in dietary knowledge and reductions in intake of sweets, soft drinks, and snacks (27)

### Cardiometabolic outcomes:

The impact on clinical biomarkers varied by intervention type and intensity. Multi-component and supervised interventions consistently produced significant improvements in weight, waist circumference, blood pressure (BP), lipid profiles, and glycemic control. Kong et al. reported significant reductions in waist circumference (−6.9 cm), systolic BP (−5.1 mmHg), total cholesterol (−23%), and fasting blood glucose (−5%) in the intervention group compared to the control group after 12 weeks (22). Schouw et al. found significant improvements in systolic BP (−10.2 mmHg), diastolic BP (−3.9 mmHg), and total cholesterol (−0.45 mmol/L) following a 24-month multi-component program (20). Shakerian et al. reported significant improvements in cardiorespiratory fitness (VO2 max:+9.99 mL/kg/min), muscular endurance (Sit-Up Test: +6.55 sit-ups), and body composition (BMI: −0.26 kg/m^2^, body fat percentage: −0.44%) after 6 months (26).

Single-component SB or PA interventions generally resulted in trivial or non-significant changes in cardiometabolic risk factors. Dunning et al. found no significant changes in BP, cholesterol, triglycerides, or glucose metabolism despite a 66 min: +9.99 mL/kg/min/day reduction in sitting time (19). Phaswana et al. reported small effect sizes for most outcomes (e.g., BMI d=−0.11; diastolic BP d=−0.26) (21).

A summary of the intervention effects on these outcomes is presented in Table 3.

### Implementation

Common challenges across studies included high participant dropout rates, often linked to the time commitment required (ranging from 15–30% in intensive programs) (22,23), external disruptions like the COVID-19 pandemic(21,31) and declining adherence to digital components over time (28). Chandrasekaran et al. reported that initial high engagement with the smartphone app (77%) declined to 31% by month 6, with qualitative findings identifying barriers including workload, lack of movement sensing, and insufficient organizational and peer support (28).

### Risk of bias assessment

**Figure 2:**
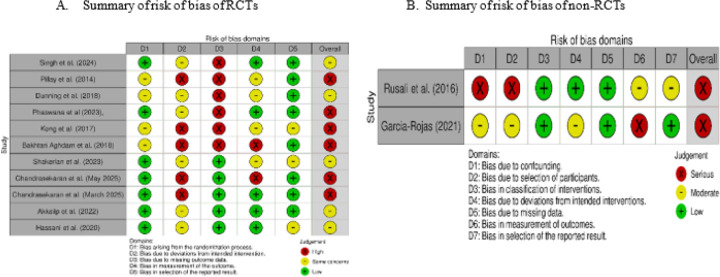
Risk of bias assessment

Assessment using Cochrane RoB 2 and ROBINS-I tools revealed moderate-to-high risk of bias across several domains (Fig. 2). Key concerns included performance bias arising from the inability to blind participants to behavioral interventions. Long-term studies reported 15%–48% attrition, which may have introduced attrition bias and influenced the magnitude of observed effects.

Measurement bias was common in studies assessing PA and dietary outcomes using self-reported questionnaires rather than device-based measures. Of the 16 studies, only 7 used device-based measures for PA or SB, while nearly 60% relied on self-reported questionnaires to measure outcomes, increasing the risk of reporting and recall bias. Confounding and selection bias were present in non-randomized studies.

## Discussion

This systematic review synthesizes evidence from 16 studies evaluating workplace interventions targeting CVD risk factors among employees in LMICs. Our findings indicate that workplace interventions can be effective in improving health-related behaviors, with multi-component, theory-informed programs showing the promise for achieving meaningful cardiometabolic benefits. However, significant challenges in sustainability, scalability, and methodological rigor persist.

### PA/SB findings

The studies using device-based PA measures revealed two important patterns. First, pedometer-based interventions can increase in daily steps when implemented at scale. This magnitude of change is clinically meaningful, as previous research suggests that an increase of 2,000 steps/day is associated with a 10% reduction in cardiovascular events (33). However, smaller pedometer-based interventions achieved modest increases (+ 300–1,000 steps/day), suggesting that intervention design, scale, and intensity matter.

Second, accelerometer-measured sedentary time reductions were consistently modest (6–66 min/day), with the largest effects observed requiring high-frequency prompting. The clinical significance of a small reduction in sedentary time is questionable, as data suggest that replacing sedentary time with light activity produces smaller cardiometabolic benefits than replacing it with MVPA(34). This may explain why SB-only interventions in this review failed to demonstrate improvements in cardiometabolic biomarkers.

Our findings are broadly consistent with systematic reviews from high-income countries (HICs), which have also reported that multi-component interventions produce the largest effects (11). However, the effect sizes observed in LMIC studies appear somewhat smaller than those reported in HIC reviews. For example, a recent meta-analysis of workplace PA interventions in HICs reported mean increases of 0.92 days of PA per week and 1,600 steps/day (35), compared to the variable 300–3,500 steps/day observed in our review. Similarly, SB reductions in HIC studies have ranged from 30–100 min/day (35) compared to 6–66 min/day in LMIC studies. This may reflect differences in intervention types, resources, or contextual factors such as workplace infrastructure and organizational support.

The pattern of SB-only interventions failing to improve clinical outcomes is consistent with HIC evidence. A systematic review and meta-analysis reported statistically significant but small effects of replacing sedentary time with PA on weight, waist circumference, body fat percentage, systolic BP, insulin, and cholesterol levels (36). However, these effects were substantially smaller than those achieved with MVPA-focused interventions. This pattern may be explained by the pathophysiology of SB: prolonged sitting rapidly induces detrimental cellular adaptations in skeletal muscle (e.g., reduced lipoprotein lipase activity) (37). Short-term interruptions in sitting may not be sufficient to alter systemic biomarkers such as fasting glucose or lipid profiles within the timeframe of typical workplace trials. This suggests that SB reduction should be viewed as a necessary but insufficient component of workplace health promotion and should be coupled with MVPA to yield measurable clinical benefits.

### The primacy of multi-component, ecologically framed interventions

Consistent with evidence from HIC (11,36), the most robust improvements in both behavioral and clinical outcomes were observed in interventions that simultaneously targeted individual, social, environmental, and organizational levels. Programs such as ‘Healthy Choices at Work’ (24) and the structured intervention by Shakerian et al.(26), which integrated education, supervised exercise, environmental modifications, and policy support demonstrated significant reductions in BP, cholesterol, and body weight. This aligns with the Socio-Ecological Model or Social Cognitive theory which posits that durable behavior change is best supported by creating reinforcing conditions across multiple levels of influence. In LMIC workplaces, where structural and cultural barriers to health may be pronounced, such holistic approaches appear particularly vital.

### The feasibility-scalability trade-off in LMIC contexts

Our review highlights a tension between intervention intensity and practical scalability in resource-constrained settings. While intensive, supervised programs produced the strongest effects, they also faced high attrition and substantial resource demands. Conversely, passive environmental strategies (e.g., canteen modifications) and low-touch digital tools demonstrated higher feasibility, albeit with modest and variable outcomes. This highlights the need for “smart scaling” strategies, i.e., designing interventions that balance evidence-based potency with real-world pragmatism, possibly through adaptive or stepped-wedge designs that tailor intensity to organizational capacity and employee need.

### Strengths and Limitations

This systematic review focused exclusively on workplace CVD prevention interventions in LMIC. Several limitations must be acknowledged. First, we cannot perform meta-analysis due to heterogeneity across studies. Second, the moderate-to-high risk of bias in many included studies, particularly due to lack of blinding and self-reported outcomes, may overestimate true effects. Third, our restriction to English-language publications may have also omitted relevant studies from our review.

## Conclusion

There is a scarcity of literature on workplace interventions focusing on CVD or lifestyle risk factors in LMIC. Despite these limitations, this review provides a foundational synthesis for a rapidly evolving field. Our findings suggest starting with feasible, low-cost environmental changes (e.g., healthy canteen options, point-of-decision prompts) as an entry point for workplace wellness and gradually layering in educational and social support components as resources allow, aiming for a multi-level approach.

Future research should prioritize: (1) development and testing of multi-component interventions explicitly designed to increase MVPA; (2) use of objective measures (e.g. accelerometers) for all behavioral outcomes; (3) hybrid effectiveness-implementation designs to assess both outcomes and contextual factors; (4) longer-term follow-up to assess sustainability; (5) and cost-effectiveness analyses.

## Conclusion

Workplace interventions in LMICs show promise for improving CVD risk profiles, particularly multi-component programs, although the number of studies remains limited. Simple modifications in physical activity, sedentary behavior and diet provide a feasible entry point but should be augmented with components designed to increase MVPA.

## Supplementary Material

Supplementary file

S1: Search strategies used for each database and results

Tables

Tables are available in the Supplementary Files section.

Supplementary Files

This is a list of supplementary files associated with this preprint. Click to download.

- Table1.png
- Table2.pdf
- Table3.png
- SupplementaryFile.docx

## Figures and Tables

**Table 1:** Population, Intervention, Comparison, Outcomes, and Study (PICOS) framework

Parameter	Inclusion criteria	Exclusion criteria
Population	Sedentary workers/employees, healthy free from diagnosed CVD, working-age adults (≥ 18 years).	Remote workers or employees working from home.
Intervention	Interventions targeted at least one CVD prevention strategy: (i) PA, (ii) sedentary behavior reduction, and/or (iii) dietary improvement	-
Comparative and context	Studies include any comparative group, such as another active intervention, passive intervention, placebo, no intervention, or waitlist control. Studies conducted in workplace LMIC settings	-
Outcome	CVD risk factors/increased PA levels/improved dietary habits/reduction in sedentary behavior cardiometabolic risk factors, cardiovascular risk factors	Focusing solely on cognitive, performance, or productivity-related outcomes without reporting PA, SB, or dietary outcomes
Study design	Randomized controlled trials (RCTs), cluster RCTs, quasi-experimental designs, pre–post design, pilot/feasibility and implementation trials	Cross-sectional studies, observational surveys, editorials, protocols without results, and interventions delivered exclusively to remote workers.

CVD: Cardiovascular diseases, PA: Physical activity, SB: Sedentary behavior
